# Acupuncture Improves Comorbid Cognitive Impairments Induced by Neuropathic Pain in Mice

**DOI:** 10.3389/fnins.2019.00995

**Published:** 2019-09-20

**Authors:** Jae-Hwan Jang, Yu-Kang Kim, Won-Mo Jung, Hyung-Kyu Kim, Eun-Mo Song, Hee-Young Kim, Ju-Young Oh, Ji-Yeun Park, Yeonhee Ryu, Mi-Yeon Song, Hi-Joon Park

**Affiliations:** ^1^Acupuncture and Meridian Science Research Center, Kyung Hee University, Seoul, South Korea; ^2^Department of Korean Medical Science, Graduate School of Korean Medicine, Kyung Hee University, Seoul, South Korea; ^3^BK21 PLUS Korean Medicine Science Center, College of Korean Medicine, Kyung Hee University, Seoul, South Korea; ^4^VUNO Inc., Seoul, South Korea; ^5^Department of Oral Physiology, School of Dentistry, Kyungpook National University, Daegu, South Korea; ^6^Department of Physical Medicine and Rehabilitation, Graduate School of Korean Medicine, Kyung Hee University, Seoul, South Korea; ^7^College of Korean Medicine, Daegu Haany University, Daegu, South Korea; ^8^College of Korean Medicine, Daejeon University, Daejeon, South Korea; ^9^Korea Institute of Oriental Medicine, Daejeon, South Korea

**Keywords:** acupuncture, partial sciatic nerve ligation, analgesia, cognitive impairment, glutamatergic receptor

## Abstract

Growing evidence indicates that neuropathic pain is frequently accompanied by cognitive impairments, which aggravate the quality of life of chronic pain patients. Here, we investigated whether acupuncture treatments can improve cognitive dysfunction as well as allodynia induced by neuropathic pain in mice. One week after the left partial sciatic nerve ligation (PSNL), acupuncture treatments on the acupoints GB30-GB34 (AP1), HT7-GV20 (AP2), or control points (CP) were performed for 4 weeks. Notably, the significant attenuations of mechanical allodynia and cognitive impairment were observed in the AP1 group, but not in PSNL, AP2, or CP groups. A random decision forest classifier based on the pain and cognitive functions displayed that the acupuncture group was clearly segregated from the other groups. We also demonstrated that acupuncture restored the reduced field excitatory post-synaptic potentials and was able to elevate the expression levels of glutamate receptors (NR2B and GluR1) in the hippocampus. Moreover, the expressions of Ca^2+^/calmodulin-dependent protein kinase II and synaptic proteins (pPSD-95 and pSyn-1) were enhanced by acupuncture treatment. These results suggest that acupuncture can enhance hippocampal long-term action through the regulation of the synaptic efficacy and that acupuncture may provide a viable option for managing both pain and cognitive functions associated with chronic neuropathic pain.

## Introduction

Chronic pain conditions are among the most common causes of disability worldwide. In addition to pain and disability, chronic pain is also associated with cognitive and emotional disorders which further diminish the quality of life (Price, 2000; Nicholson and Verma, 2004; Guida et al., 2015; De Gregorio et al., 2019). Current pharmacological treatments often do not meet patients’ needs due to unsatisfactory efficacy and adverse effects. In addition, the comorbidities may cause patients to seek multiple treatments, increasing their financial burdens. Thus, it is essential to enhance therapeutic outcomes by taking a holistic approach targeting these multidimensional aspects of chronic pain while controlling for side effects (Bergbom et al., 2011; Hopton et al., 2014).

To date, human studies have indicated that supraspinal structures including the hippocampus, anterior cingulate cortex, medial prefrontal cortex, and dorsal raphe nucleus are involved in chronic pain (Fields et al., 1977; Wang and Nakai, 1994; Valet et al., 2009; Zimmerman et al., 2009; Khan et al., 2014). In particular, the reduced hippocampal volume and changes in hippocampal structures were found in patients with chronic pain, implicating pain-related cognitive dysfunction (Valet et al., 2009; Zimmerman et al., 2009; Ezzati et al., 2014). Many studies have also shown the functional impairment of hippocampus including abnormal cytokine expression, short-term and working memory deficits as well as impairment of long-term potentiation (LTP) in animal models of chronic pain (Kodama et al., 2007, 2011; Ren et al., 2011; Wu et al., 2015).

Nerve injuries also affect synaptic plasticity and induce numerous changes in multiple neurotransmitters and intracellular signal transduction through changes in the expression or function of excitatory and inhibitory transmissions in the hippocampus (Mutso et al., 2012; Wang X.Q. et al., 2015; Liu et al., 2017; Saffarpour et al., 2017). Glutamate is a major excitatory neurotransmitter of the central nervous system, and recent studies have investigated the potential roles of the hippocampal glutamatergic system in the pathophysiology of pain (Wang X.Q. et al., 2015; Saffarpour et al., 2017). It is widely recognized that glutamate regulates excitatory synaptic transmission in the hippocampus via the NMDA receptor (NMDAR) and α-amino-3-hydroxy5-methyl-4-isoxazolpropionic acid receptor (AMPAR). An NMDAR subunit, NR2B, is involved in various physiological processes including learning, memory, and synaptic plasticity by regulation of LTP induction. In addition, calcium/calmodulin-dependent protein kinase II (CaMKII) has a critical role in LTP induction. Reduced levels of glutamate receptors and CaMKII were found in the hippocampal area of the neuropathic pain model (Xu et al., 2012).

Both clinical and animal studies have shown that acupuncture significantly improves chronic pain (Kim et al., 2005, 2007). In addition, acupuncture could mitigate pain-related comorbidities such as depression and insomnia in chronic pain patients (Huang et al., 2011; Hopton et al., 2014). Several animal studies showed that acupuncture improves both the nociceptive and cognition-related behaviors in a cobra venom-induced chronic neuropathic pain model (Chen et al., 2017). However, it remains elusive how acupuncture rescues comorbid conditions as well as pain simultaneously.

In the present study, we first examined whether acupuncture could improve both cognitive and pain behaviors in the partial sciatic nerve ligation (PSNL)-induced neuropathic pain model. Next, a random decision forest classifier in machine learning was used to see if the therapeutic effects of acupuncture can be predicted based on pain and cognitive behaviors. Then, we examined the changes in glutamatergic receptors and synaptic proteins as well as LTP in the hippocampus to elucidate the molecular mechanism of therapeutic effects by acupuncture.

## Materials and Methods

### Animals

Seven-week-old male C57BL/6 mice (22 to 25 g in body weight; Samtaco, Seoul, South Korea) and four-week-old male C57BL/6 mice (for patch clamp recording; Samtaco) were individually housed at 24 ± 2°C under a 12/12 h light/dark cycle (light: 08:00 to 20:00, dark: 20:00 to 08:00) for at least 7 days before conducting experiments with free access to food and water.

### Induction of Neuropathic Pain by PSNL

A left hind paw PSNL model was established in accordance with the approach by Malmberg and Basbaum (1998) with a few modifications. Mice were anesthetized with rompun (100 μl, intraperitoneally (i.p.); Bayer, Seoul, South Korea) and 2% zoletil (100 μl, i.p.; Virbac S.A., Carros, France). The bilateral hind thigh was shaved, and the sciatic nerve was exposed using scissors. Then, the dorsal 1/3 to 1/2 of the nerve was lightly ligated with 8-0 silk (AILEE, Busan, South Korea), and the open wound was closed. In non-nerve injured group (Sham; *n* = 10), the nerve was exposed without injury and was closed. Seven days after PSNL surgery, mice were randomly assigned to one of 5 groups: PSNL, acupuncture 1 (AP1), acupuncture 2 (AP2), control points (CP), or amitriptyline groups (each *n* = 10).

### Acupuncture and Control Treatments

**FIGURE 1:**
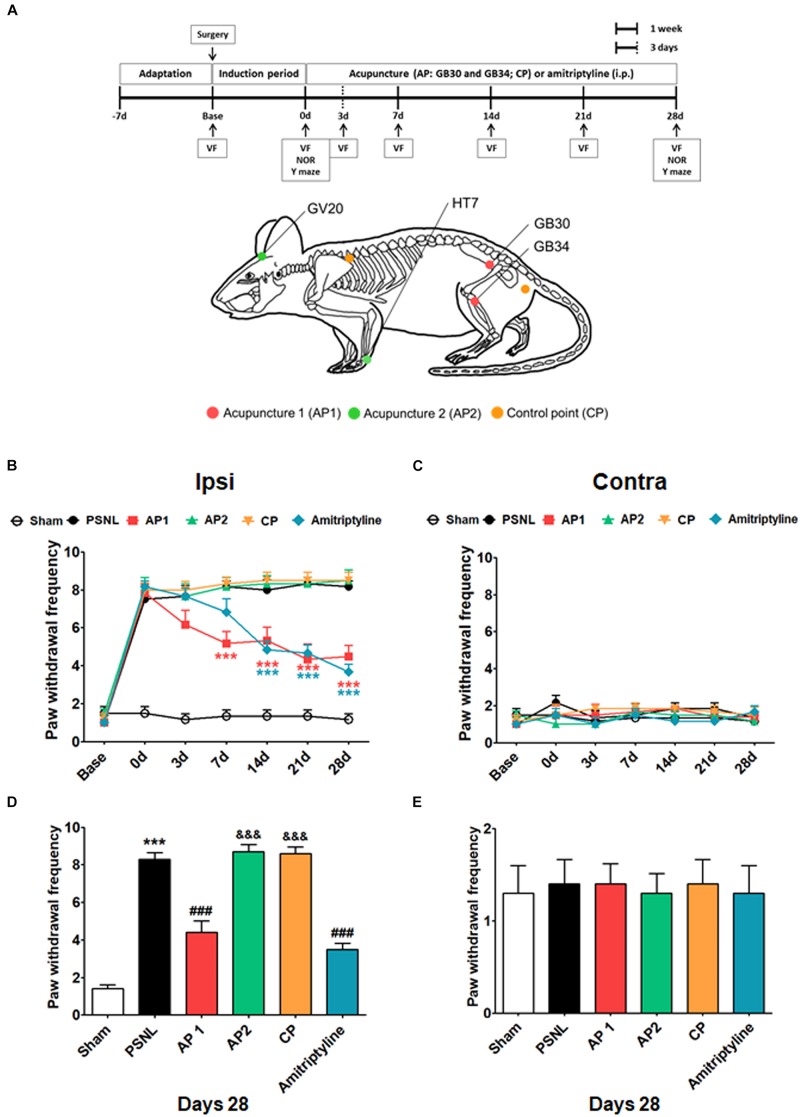
Effects of acupuncture on mechanical allodynia evaluated through von Fey test. Experimental design of acupuncture (AP1, AP2, or CP) or amitriptyline (10 mg/kg, i.p.) administration in the PSNL-induced neuropathic pain model. Locations of the acupuncture treatment GB30 (Hwando) and GB34 (Yanglingquan) [AP1 (red circle)], HT7 (Sinmun) and GV20 (Baekhoe) [AP2 (green circle); dapple green circle indicated backside], and control point [CP (orange circle)] **(A)**. ^∗∗∗^*p* < 0.001 vs. the PSNL group. Anti-allodynic effects of acupuncture (AP1, AP2, or CP) or amitriptyline was measured on the ipsilateral **(B,D)** and contralateral **(C,E)** plantar surfaces 2 h after the administration (for 0, 3, 7, 14, 21, and 28 days). *n* = 10/group. ^∗∗∗^*p* < 0.001 vs. the Sham group, ^###^*p* < 0.001 vs. the PSNL group, and ^&⁣&⁣&^*p* < 0.001 vs. the AP1 group. Data were analyzed with a two-way repeated measures ANOVA followed by *post hoc* Bonferroni tests. The results are expressed as the mean ± SEM.

Acupuncture treatments were performed at acupoints GB30 and GB34 (AP1 group), HT7 and GV20 (AP2 group), or two control points (CP group) bilaterally, which continued daily for 28 consecutive days starting from day 7 after surgery (Figure 1A). GB30 (at the junction of the lateral 1/3 and medial 2/3 of the greater trochanter with the sacral hiatus) and GB34 (in the depression anterior and distal to the head of the fibula) are combinations of acupoints that are utilized for the treatment of lumber neuropathic pain in the clinic (Cho et al., 2013; Ju et al., 2017). HT7 (radial to the flexor carpi ulnaris tendon on the palmar crease) and GV20 (on the vertex of the head) are combinations of acupoints frequently used for the treatment of cognitive and emotional impairments (Lee et al., 2012; Chen et al., 2016; Fleckenstein et al., 2018; Yu et al., 2018). The detailed locations of acupoints were shown in Figure 1A. For the acupuncture treatments, mice were mildly immobilized, and a sterilized acupuncture needle (8 mm in length and 0.18 mm in diameter; Haenglim-seoweon Acuneedle Co., South Korea) was inserted bilaterally to a depth of 3 mm and turned bi-directionally for 30 s at a rate of two spins per second, one 180° clockwise rotation and 180° counterclockwise rotation, by bare fingers. In order to rule out the non-specific effects of acupuncture, the same acupuncture stimulation was given to control points (CP group). The two control points were located at the non-acupoints, approximately 3 mm lateral from the middle of the medial border of the scapula or the tail base on the gluteus muscle (Figure 1A).

Amitriptyline is a tricyclic antidepressant, and is widely used to treat various neuropathic pain (Max et al., 1987; Benbouzid et al., 2008; Palazzo et al., 2016). As a positive control, amitriptyline (amitriptyline group; 10 mg/kg in 100 μl, Sigma-Aldrich, St. Louis, MO, United States) was given i.p. daily for 28 consecutive days from day 7 after the PSNL surgery. A solution of amitriptyline was freshly prepared on each treatment day in 0.9% NaCl (Berrocoso et al., 2011). To subject animals to the equal stress condition, the Sham, PSNL and amitriptyline group animals were also mildly immobilized as done in AP and CP groups.

### Nociceptive Behavior Test

The electronic von Frey test (IITC, Woodland Hills, CA, United States) was conducted (Jang et al., 2018) to evaluate the mechanical allodynia. This test was performed before surgery and treatment (Base and 0 day), respectively, and was also conducted at 3, 7, 14, 21, and 28 days after 2 h of acupuncture and amitriptyline treatment (AM 11:00–PM 06:00).

### Cognitive Function Tests

Spatial working memory was measured by spontaneous alternation behavior in Y-Maze. Each mouse was placed in one of the Y-maze arms and allowed to explore freely through the maze during a 5-min session (Palazzo et al., 2016). The sequence and the total number of arms entered were recorded. An arm entry was considered complete when both hind paws were in the arm. The apparatus was cleaned with water and ethanol between each passage. Percentage of spontaneous alternation was determined by the number of triads containing entries into all three arms/maximum possible alternations (total number of arms entered − 2) × 100 (Palazzo et al., 2016).

The novel object recognition (NOR) test was used to evaluate the short-term memory. The apparatus consisted in a 40 × 40 × 27 cm acrylic box with white walls and floor. The box and objects were cleaned between trials to eliminate olfactory cues. Animals received 5 min sessions in the empty box for habituation to the apparatus and test room (Palazzo et al., 2016). Twenty-four hours later, each mouse was exposed to two familiar objects (block of round, diameter: 4 cm) during 5 min “training stage” in the box. Next, the animal was placed back in the box and exposed to a novel object (block of rectangle, 4 × 4 × 4 cm) as well as the familiar object for another 5-min “test stage” at 24 h after “training stage.” The time spent exploring each object was measured. The recognition index reflecting the short-term memory ability was calculated as the ratio of time spent exploring the novel object over total exploration time (Palazzo et al., 2016). All cognitive function tests were performed before treatment (AP1, AP2, CP, or amitriptyline) and on day 28 of treatment. The tests were conducted from 2 h after treatment (AM 11:00–PM 06:00).

### A Random Decision Forest Classifier in Machine Learning

Based on the three behavioral data (mechanical allodynia, Y-maze, and NOR), a random forest classifier was used to test whether we could predict which mouse belongs to which experimental group. Random forest classifier is an ensemble method that incoporates decision tree models with multiple randomness and predicts them through the average value of the predictions of these trees (Breiman, 2001; Geurts et al., 2006). The classifier of this study was analyzed for three types of behavioral data that were labeled by the experimental group using Python’s scikit learn package^1^ (Pedregosa et al., 2011). The classification accuracy was obtained by applying fourfold cross validation. One hundred iterations were performed to extract the average value. In addition, we randomly permutated the labeling of the experimental group by repeating the procedure 10,000 times to generate a statistical null model for comparison. In order to express the process and meaning of classification, a decision tree was obtained at four depth levels. Finally, mouse data were represented on a scatter plot using two pairs of measurements for pain and cognitive function.

### Western Blotting

After anesthetized, the brain was extracted. Brain tissue samples including the hippocampus were homogenized in 200 μL of lysis buffer, containing 20 mM hydroxyethyl piperazineethanesulfonic acid (pH 7.5), 1% NP-40, 10% glycerol, 150 mM NaCl, 60 mM B-Glucoside, 1 mM phenylmethanesulfonyl fluoride, 0.7 μg/mL Pepstatin, phosphatase and protease inhibitor cocktail tablets. Western blot was performed (Park et al., 2014) to measure the protein expression levels of glutamatergic receptors and synaptic proteins in the hippocampus. Primary antibodies were rabbit anti-phospho-NR2B (pNR2B), rabbit anti-total-NR2B (tNR2B) (diluted 1:1,000; Merck Millipore, Darmstadt, Germany), rabbit anti-phospho-GluR1 (pGluR1), rabbit anti-total-GluR1 (tGluR1), rabbit anti-phospho-CaMKII (pCaMKII), rabbit anti-total-CaMKII (tCaMKII), rabbit anti-phospho-protein kinase C-γ (pPKC-γ), rabbit anti-total- protein kinase C-γ (pPKC-γ), rabbit anti-phospho-Syn-1 (pSyn-1), rabbit anti-total-Syn-1 (tSyn-1), rabbit anti-phospho-PSD-95 (pPSD-95), rabbit anti-total-PSD-95 (tPSD-95) (diluted 1:1,000; Cell Signaling Technology, Beverly, MA, United States), and rabbit anti-β-actin (diluted 1:10,000; Sigma-Aldrich). Then, the membrane was incubated with the secondary horseradish peroxidase-conjugated goat anti-rabbit antibody (diluted 1:1,000; Pierce, Rockford, IL, United States). The membrane was visualized using a chemiluminescence kit (Super Signal West Pico; Pierce), and the signal intensities were analyzed by a densitometry and image QI software. We show all bands before contrast modification (Supplementary Figure 4).

### Immunofluorescence

Immunofluorescence was conducted (Jang et al., 2018) to measure the expression of NR2B and GluR1 in neuron cells in the PFC. Primary antibodies raised against NeuN (mouse, 1:500, MAB377; Chemicon International, Inc., Temecula, CA, United States), GFAP (mouse, 1:500, 14-9892-82; Thermo Fisher Scientific, San Diego, CA, United States), Iba-1 (mouse, 1:500, MABN92; Merck Millipore), NR2B (rabbit, 1:500, 06-600; Merck Millipore), and GluR1 (rabbit, 1:1000, #8084; Cell Signaling Technology) were diluted in 1 × PBST supplemented with 0.1% BSA. The incubation was performed in dark at 4°C for 72 h. Next, following PBST washes, the sections were incubated for 1 h with a mixture of Alexa 488-conjugated donkey anti-rabbit secondary antibody (1:1000; Thermo Fisher Scientific) and Alexa 594-conjugated donkey anti-mouse secondary antibody (1:1000; Thermo Fisher Scientific). The numbers of NeuN/NR2B and NeuN/GluR1 double-positive cells within the CA1, CA3, and DG in the hippocampus were counted three times by a researcher blind to each group using a square grid (300 × 300 μm). The mean counts were defined as the numbers of NeuN/NR2B and NeuN/GluR1 double-positive cells. Additionally, tissues incubated without primary antibody were used as a negative control (Supplementary Figure 3).

### Electrophysiology

After post-surgery day 35, electrophysiological recordings were made. The electrophysiology was performed (Citri and Malenka, 2008; Villers and Ris, 2013; Sweet et al., 2015) to measure LTP. Following isoflurane anesthesia, the brain was removed, and the hippocampus was quickly dissected out with a vibratome (VT 1200 S; Leica Microsystems, Wetzlar, Germany). Hippocampal slices (400 μm) were incubated in 20 mL artificial cerebrospinal fluid (aCSF; containing 119 mM NaCl, 2.5 mM KCl, 1 mM NaH_2_PO_4_, 26.2 mM NaHCO_3_, 11 mM D-glucose, 2.5 mM CaCl_2_, and 9 mM MgCl_2_; pH = 7.2–7.4; 4°C) saturated with 5% CO_2_ in O_2_ at 32°C for at least 1 h. Then, the prepared slices were transferred to a recording chamber, containing oxygenated aCSF (119 mM NaCl, 2.5 mM KCl, 1 mM NaH_2_PO_4_, 26.2 mM NaHCO_3_, 11 mM D-glucose, 2.5 mM CaCl_2_, 1.3 mM MgCl_2,_ and 0.4 mM ascorbic acid; pH = 7.2–7.4; 4°C) at a flow rate of 2 mL/min at 32°C. Glass electrode (recording electrode) were filled with recording aCSF and placed in the stratum radiatum of CA1. Electrodes were lowered 75 to 150 μm under the surface of the slice using micromanipulator (ROE-200, Sutter Instruments, Novarto, CA, United States) and Controller (MPC-200, Sutter Instruments). Then, a bipolar electrode (Stimulation electrode) was placed in the CA3 area to evoke the field excitatory postsynaptic potential (fEPSP) responses (Citri and Malenka, 2008; Sweet et al., 2015). The hippocampal slices were stimulated with 0.5 mV/0.5 ms every 30 s for at least 10 min, followed by 0.5 mV/100 Hz/1 s for 15 min of test stimulation. This signal amplified by an amplifier (MultiClamp 700B, Molecular Devices) was then sent to a computer through A/D converter (Digidata 1440A, Molecular Devices, Sunnyvale, CA, United States) and analyzed using Clampex 10.7 Software (Molecular Devices).

### Statistical Analysis

All statistical parameters were calculated using GraphPad Prism 5.0 software (GraphPad Software, San Diego, CA, United States). An unpaired two-tailed *t*-test was used for comparing the difference in emotional and cognitive impairment between sham and PSNL groups. Cognitive impairment behaviors based on the Y-maze and NOR test, electrophysiology, western blot and immunohistochemical data were subjected to one-way analysis of variance (ANOVA) tests followed by Newman–Keuls tests. Analyses of mechanical allodynia at various time points were performed using two-way repeated measures ANOVAs and Bonferroni *post hoc* tests for pairwise multiple comparisons. Spearman rank correlation coefficient tests were conducted to analyze whether the von Frey test was correlated with Y-maze and NOR test, and whether the expression levels in NR2B and GluR1 in the hippocampus were correlated with von Frey and NOR test. All data are expressed as the mean ± standard error of the mean (SEM). For all analyses, *p* < 0.05 was considered to indicate statistical significance.

## Results

### Acupuncture Improves Mechanical Allodynia in PSNL-Induced Neuropathic Pain

The anti-allodynic and analgesic effects of acupuncture were measured using the von Frey test in the PSNL-induced neuropathic pain model. The baseline measurement was performed 1 day before PSNL surgery. Then, mice were randomly assigned into five groups. Paw withdrawal frequency in five groups did not show any difference. The effects of treatments with acupuncture (AP1, AP2, or CP) or amitriptyline (10 mg/kg, i.p.) were investigated for 28 days in the PSNL-induced neuropathic pain model (Figure 1). For mechanical allodynia, a two-way repeated measures ANOVA revealed a significant effect of group (*F*_5,246_ = 175.5, *p* < 0.0001) and a significant group × time interaction (*F*_30,246_ = 8.733, *p* < 0.0001). Bonferroni *post hoc* tests showed that AP1 treatment reversed the established ipsilateral mechanical allodynia at each time point (each *p* < 0.001 vs. PSNL over day 7 after treatment; Figure 1B). Likewise, the paw withdrawal frequency was significantly lower in the amitriptyline group than in the PSNL group (each *p* < 0.001 vs. PSNL over day 14 after treatment; Figure 1B). We also observed the effects of acupuncture (AP1, AP2 or CP) or amitriptyline administration in the nociceptive behavior test at day 28. A one-way ANOVA indicated a significant difference between groups for paw withdrawal frequency on day 28 (*F*_5,54_ = 68.08, *p* < 0.0001). Newman–Keuls *post hoc* tests revealed that the paw withdrawal frequencies were lower in the AP1 (*p* < 0.001) and amitriptyline groups (*p* < 0.001) than the PSNL group (Figure 1D). There were no significant changes in the pain levels in the contralateral hind paws (Figures 1C,E). Our results showed that acupuncture treatment in AP1 had an analgesic effect.

### The Effect of Acupuncture on Cognitive Function in PSNL-Induced Pain Model

**FIGURE 2:**
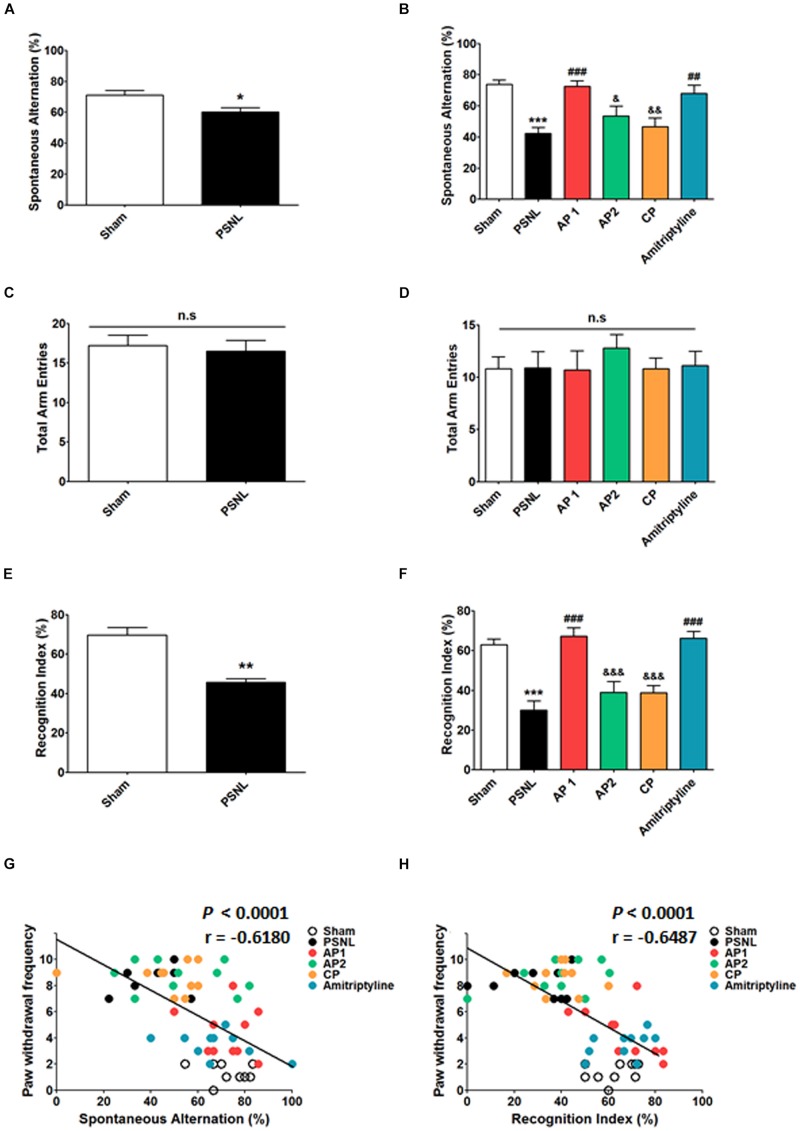
Effects of acupuncture on cognitive functions measured through Y-maze and NOR tests. Effects of acupuncture (AP1, AP2, or CP) or amitriptyline (10 mg/kg, i.p.) treatment on cognitive impairment were investigated using Y-maze **(A–D)** and NOR **(E,F)** tests in the PSNL-induced neuropathic pain model. All groups: *n* = 10. ^∗^*p* < 0.05, ^∗^*p* < 0.01, ^∗∗∗^*p* < 0.001 vs. the Sham group, ^#^*p* < 0.05, ^##^*p* < 0.01, ^###^*p* < 0.001 vs. the PSNL group, ^&^*p* < 0.05, ^&⁣&^*p* < 0.01, and ^&⁣&⁣&^*p* < 0.001 vs. the AP1 group. The data were analyzed with a one-way ANOVA followed by Newman–Keuls *post hoc* tests **(A–F)**. Data are expressed as the mean ± SEM. The cognitive function measures in both Y-maze and NOR tests were correlated with paw withdrawal frequency **(G,H)**. The *r*-values were analyzed with the Spearman rank correlation coefficient. AP1, acupuncture 1 (GB30 and GB34); AP2, acupuncture 2 (HT7 and GV20); CP, control point; NOR, novel object recognition.

To examine the effects of acupuncture treatment on PSNL-induced cognitive impairment, we used Y-maze and NOR test (Figure 2). First, we observed cognitive impairment 8 days after PSNL surgery. Unpaired two-tailed *t*-tests revealed that the spontaneous alternation in PSNL mice was significantly lower than that in sham mice (*t*_18_ = 1.120, *p* = 0.0176; Figure 2A). In addition, unpaired two-tailed *t*-tests showed that the recognition index in the PSNL group was significantly decreased by PSNL surgery (*t*_18_ = 12.20, *p* = 0.0087; Figure 2E). Next, acupuncture (AP1, AP2, or CP) or amitriptyline (10 mg/kg, i.p.) treatment was continued for 28 consecutive days and the effects of treatments on cognitive impairment were measured again at day 28. In the Y-maze test, one-way ANOVA revealed a significant difference between groups for spontaneous alternation (*F*_5,54_ = 8.450, *p* < 0.0001), and Newman–Keuls *post hoc* tests showed that spontaneous alternation in AP1 group was higher than that in the PSNL group (*p* < 0.05; Figure 2B). There were no significant changes in total arm entries of Y-maze test (Figures 2C,D). In the NOR test, a one-way ANOVA (*F*_5,54_ = 15.78, *p* < 0.0001) followed by Newman–Keuls *post hoc* tests showed that the PSNL-induced reduction in recognition index (*p* < 0.001 vs. Sham group) was restored by AP1 and amitriptyline treatments (each *p* < 0.001 vs. PSNL group), whereas AP2 and CP had no effects (Figure 2F). Furthermore, spontaneous alternation and recognition index were negatively correlated with paw withdrawal frequency (*r* = −0.6180, *p* < 0.0001; *r* = −0.6487, *p* = 0.0026, respectively; Figures 2G,H). Therefore, our results showed that cognitive functions are impaired by induced neuropathic pain and that acupuncture has the potential to improve the cognitive impairment in neuropathic pain.

### Effects of Acupuncture on Analgesia and Cognitive Function Using Machine Learning

**FIGURE 3:**
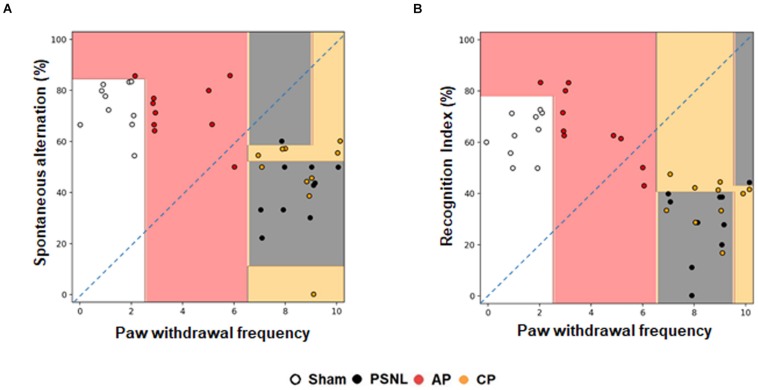
Effects of acupuncture on classification using a random forest classifier analysis in machine learning method. Cognitive functions and nociceptive behavior data of the four groups (Sham, PSNL, AP1, and CP) were analyzed by random forest algorithm in machine learning. Representative figures show that the spontaneous alternation in Y-maze **(A)** and recognition index in NOR test **(B)** were analyzed against the paw withdrawal frequency in the von Frey test. All groups: *n* = 10. AP1, acupuncture 1 (GB30 and GB34); CP, control point.

The decision tree algorithm consists of a random forest algorithm that is internally trained to express the distribution of behavior data values, the classification process, and semantics for each experimental group. It displays the decision boundary of the entire 40 mouse data on the scatter plot. This is presented in Figure 3. The Sham and AP1 groups were segregated from the PSNL and CP groups. The Sham and AP1 groups showed lower pain responses, while the PSNL and CP groups showed higher pain responses. The pain response data appear to provide useful information in distinguishing the Sham and AP1 groups, while the NOR and Y-maze data do not. The pain data between the PSNL and CP groups do not differ significantly, but the Y-maze and NOR data apparently allow a better distinction between them (Figures 3A,B). By applying 4-fold cross validation, we predicted with a 64.4% accuracy the membership of each mouse in each experimental group through the three threshold values of pain, NOR, and Y-maze.

### The Effects of Acupuncture on LTP From CA3 to CA1 Regions of the Hippocampus in PSNL

**FIGURE 4:**
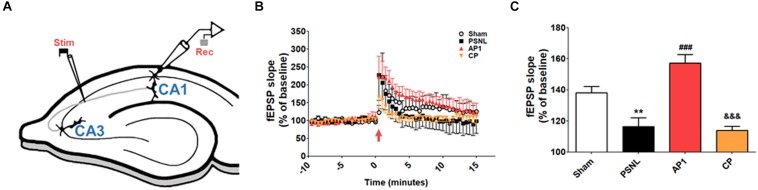
Acupuncture increased fEPSP slopes in the hippocampal CA3 to CA1 areas of neuropathic pain model. A schematic of a transverse hippocampal area used in electrophysiology methods, showing recording (rec) and stimulating (stim) regions **(A)**. Averaged fEPSP data. High-frequency stimulation was given at 0 min, and the signal was followed for up to 15 min **(B)**. Summary of fEPSP slopes (from 0 to 15 min) shown in **(C)**. *n* = 5 in all groups. ^∗∗^*p* < 0.01 vs. the Sham groups. ^###^*p* < 0.001 compared with the PSNL group, and ^&⁣&⁣&^*p* < 0.001 vs. AP1 group. The data were analyzed with a one-way ANOVA followed by Newman–Keuls *post hoc*tests. The results are expressed as the mean ± SEM. AP1, acupuncture 1 (GB30 and GB34); AP2, acupuncture 2 (HT7 and GV20); CP, control point.

LTP recording was performed to investigate the electrophysiological basis of the effects of acupuncture (Figure 4). After 14–28 days of acupuncture treatment (AP1), the time course of fEPSP slopes that were normalized to the 15 min baseline period was presented in Figure 4B. The fEPSP slopes of the four groups were increased immediately after electronic stimulation and stabilized to different levels above the baseline. The statistical mean value of the last 15 min was shown in Figure 4C. One-way ANOVA showed that there were significant differences in the mean slopes of fEPSP among the four groups (*F*_3,120_ = 19.17, *p* < 0.001, Figure 4C). Newman–Keuls *post hoc* tests showed that the mean fEPSP slopes were smaller in the PSNL and CP groups than in the Sham (*p* < 0.01 vs. PSNL) and AP1 groups (*p* < 0.001 vs. PSNL group; *p* < 0.001 vs. CP group), indicating that acupuncture treatment did efficiently reverse the PSNL-induced LTP impairment.

### Effects of Acupuncture on Expression Levels of Glutamate Receptors in the Hippocampus of PSNL Mice

**FIGURE 5:**
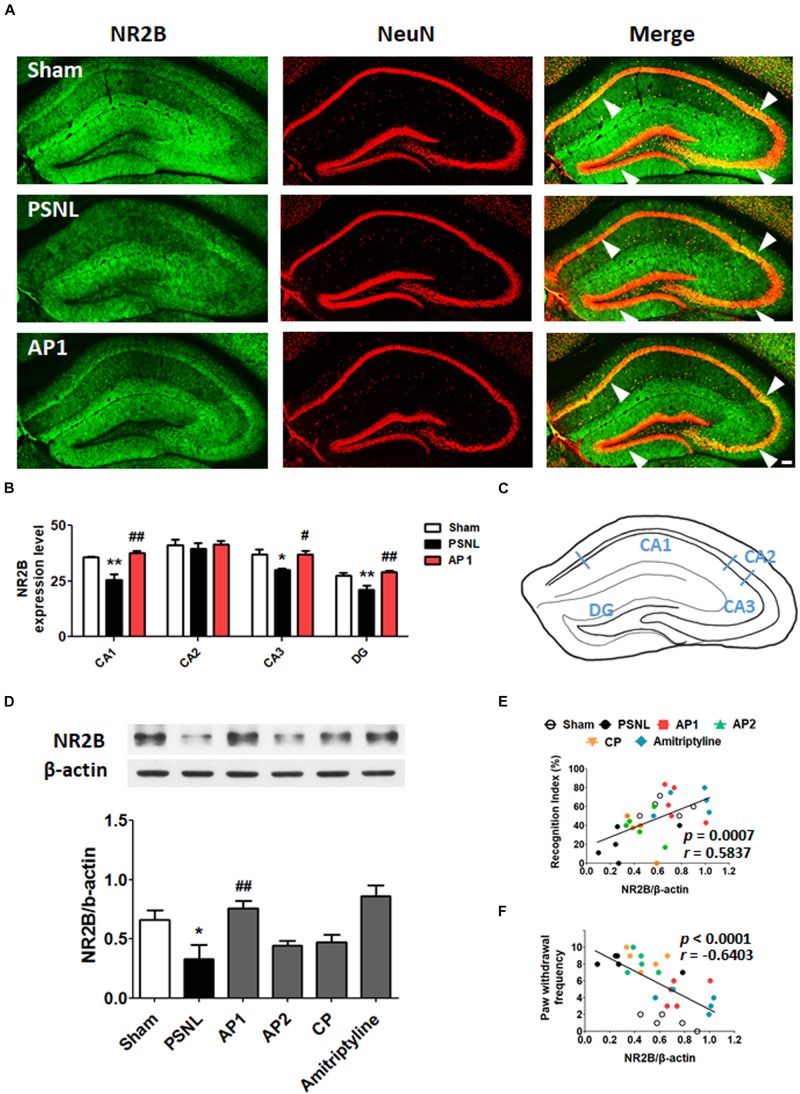
Effects of acupuncture on the expression levels of hippocampal NR2B receptor. These results show the changes in hippocampal NR2B expression levels (CA1, CA2, CA3, and DG) after administration of acupuncture (AP1, AP2, or CP) or amitriptyline (10 mg/kg, i.p.) for 28 consecutive days **(A–F)**. Histological examinations of the hippocampus showing the expression of NR2B (green) and NeuN (red) after AP1 administration in the PSNL-induced neuropathic pain model **(A,B)** and a representative figure showing the hippocampal regions in the mouse brain **(C)**. *n* = 3–4/group. Scale bar: 100 μm. ^∗^*p* < 0.05, ^∗∗^*p* < 0.01 compared to the Sham group in each area. ^#^*p* < 0.05, ^##^*p* < 0.01 compared to the PSNL group in each area. The NR2B protein levels were measured in the hippocampus **(D)**. *n* = 5/group. ^∗∗^*p* < 0.01 compared to the Sham group. ^#^*p* < 0.05 compared to the PSNL group. The results were analyzed with a one-way ANOVA followed by Newman–Keuls *post hoc* tests. The data are expressed as the mean ± SEM. The cognitive function values in the NOR test and nociceptive values in the von Frey test were correlated with the hippocampal NR2B protein levels **(E,F)**. The *r*-values were analyzed with the Spearman rank correlation coefficient. AP1, acupuncture 1 (GB30 and GB34); AP2, acupuncture 2 (HT7 and GV20); CP, control point.

**FIGURE 6:**
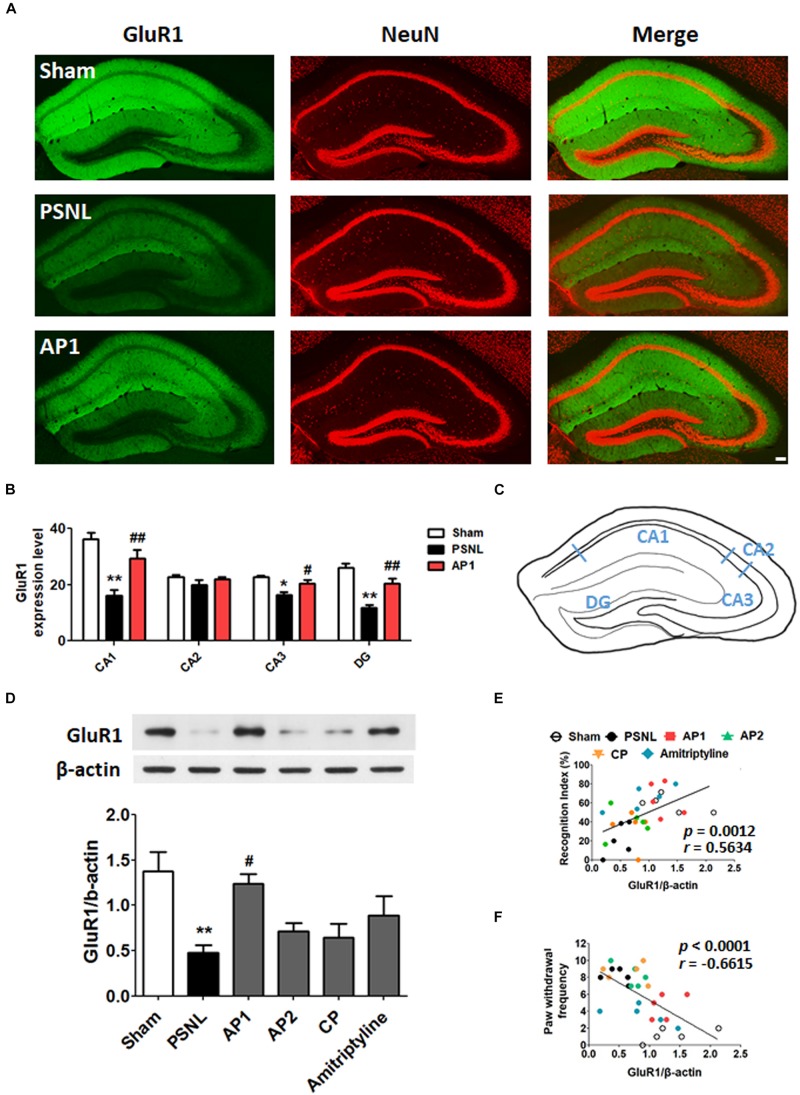
Effects of acupuncture on the expression levels of hippocampal GluR1 receptor. These results show the changes in hippocampal GluR1 expression levels (CA1, CA2, CA3, and DG) after administration of acupuncture (AP1, AP2, or CP) or amitriptyline (10 mg/kg, i.p.) for 28 consecutive days **(A–F)**. Free-floating coronal hippocampus sections from the three groups (Sham, PSNL, and AP1) were subjected to immunofluorescence with GluR1 (green) and NeuN (red) antibodies to label GluR1-positive NeuN in neurons **(A)**. Representative graphs showing the expression levels of GluR1 in the hippocampus **(A,B)** and a representative figure showing the hippocampal regions in the mouse brain **(C)**. *n* = 3–4/group. Scale bar: 100 μm. ^∗^*p* < 0.05, ^∗∗^*p* < 0.01 compared to the Sham group in each area. ^#^*p* < 0.05, ^##^*p* < 0.01 compared to the PSNL group in each area. The expression levels of hippocampal GluR1 protein were examined **(D)**. *n* = 5/group. ^∗∗^*p* < 0.01 compared to the Sham group. ^#^*p* < 0.05 compared to the PSNL group. All data were analyzed with a one-way ANOVA followed by Newman–Keuls *post hoc* tests. All data are expressed as the mean ± SEM. The cognitive function values in the NOR test and nociceptive values in the von Frey test were correlated with the GluR1 protein levels in the hippocampus **(E,F)**. The *r*-values were analyzed with the Spearman rank correlation coefficient. AP1, acupuncture 1 (GB30 and GB34); AP2, acupuncture 2 (HT7 and GV20); CP, control point.

Many studies have shown that NR2B and GluR1 plays important roles in the synaptic plasticity through induction and maintenance of LTP at the Schaffer collateral-CA1 synapses; LTP is important in learning and memory functions (Lisman et al., 2002; Fonseca, 2012; Bliss and Collingridge, 2013; Wang H. et al., 2015; Shang et al., 2017). To investigate the role of glutamate receptors in neuropathic pain, we examined hippocampal NR2B and GluR1 using double-immunostaining (Figures 5, 6). First, NR2B- and GluR1-positive cells were double stained for three cell type markers (GFAP, Iba-1 or NeuN). The results suggest that they are expressed in neurons, but not in astrocytes or microglia (Supplementary Figure 1). Next, double-immunostaining and western blotting were carried on hippocampal NR2B and GluR1 for acupuncture groups (AP1, AP2 or CP) or amitriptyline group (10 mg/kg, i.p.). Coronal sections of the hippocampus from the three groups (Sham, PSNL and AP1) were subjected to NR2B and NeuN antibodies for double-immunostaining. A one-way ANOVA showed a significant difference between the groups for expression of NR2B (CA1: *F*_2,7_ = 18.26, *p* = 0.0017; CA2: *F*_2,7_ = 0.2718, *p* = 0.7697; CA3: *F*_2,7_ = 6.523, *p* = 0.0252; DG: *F*_2,7_ = 11.23, *p* = 0.0065), and Newman–Keuls *post hoc* tests showed that expression levels of NR2B were lower in the PSNL group than those in the Sham group (CA1: *p* < 0.01, CA3: *p* < 0.05, DG: *p* < 0.01). AP1 administration reversed the attenuation of hippocampal NR2B expression resulted from the PSNL surgery (CA1: *p* < 0.01, CA3: *p* < 0.05, DG: *p* < 0.01 vs. PSNL; Figures 5A,B). In western blot analysis, one-way ANOVA showed a significant difference in protein expression of NR2B in the hippocampus between the groups (*F*_5,24_ = 6.521, *p* = 0.0006). Newman–Keuls *post hoc* tests showed that hippocampal NR2B expression levels in the AP1 group were significantly higher than those in the PSNL group (*p* < 0.01; Figure 5D). There was a positive correlation between increased expression levels of NR2B in the hippocampus and the recognition index in NOR test (*r* = 0.5837 and *p* = 0.0007; Figure 5E), but a negative correlation between levels of NR2B in the hippocampus and paw withdrawal frequency in von Frey test. (*r* = −0.6403 and *p* < 0.0001; Figure 5F).

Next, coronal sections of the hippocampus from the three groups (Sham, PSNL, and AP1) were subjected to GluR1 and NeuN antibodies. A one-way ANOVA showed a significant difference in the expression of GluR1 between the groups (CA1: *F*_2,7_ = 13.27, *p* = 0.0042; CA2: *F*_2,7_ = 1.900, *p* = 0.2192; CA3: *F*_2,7_ = 6.630, *p* = 0.0242; DG: *F*_2,7_ = 17.98, *p* = 0.0017). Newman–Keuls *post hoc* tests showed that GluR1 expression levels were lower in the PSNL group than those in the Sham group (CA1: *p* < 0.01, CA3: *p* < 0.05, DG: *p* < 0.01 vs. PSNL). Similar to NR2B, AP1 administration suppressed the reduction of hippocampal GluR1 expression levels induced by sciatic nerve surgery (CA1: *p* < 0.01, CA3: *p* < 0.05, DG: *p* < 0.01 vs. PSNL; Figures 6A,B). In western blot analysis, a one-way ANOVA showed a significant difference in protein expression of GluR1 between the groups (*F*_5,24_ = 5.216, *p* = 0.0022). Newman–Keuls *post hoc* tests showed that hippocampal GluR1 expression levels were significantly increased in the AP1 group compared to the PSNL group (*p* < 0.01; Figure 6D).

The GluR1 expression levels in the hippocampus were positively correlated with the recognition index in NOR test (*r* = 0.5634 and *p* = 0.0012; Figure 6E), and were negatively correlated with the paw withdrawal frequency in von Frey test (*r* = −0.6615 and *p* < 0.0001; Figure 6F). These results suggest that acupuncture administration may enhance cognitive functions by restoring glutamate receptors in the hippocampus in neuropathic pain.

### Effects of Acupuncture on Expression Levels of CaMKII in the Hippocampus in PSNL Mice

**FIGURE 7:**
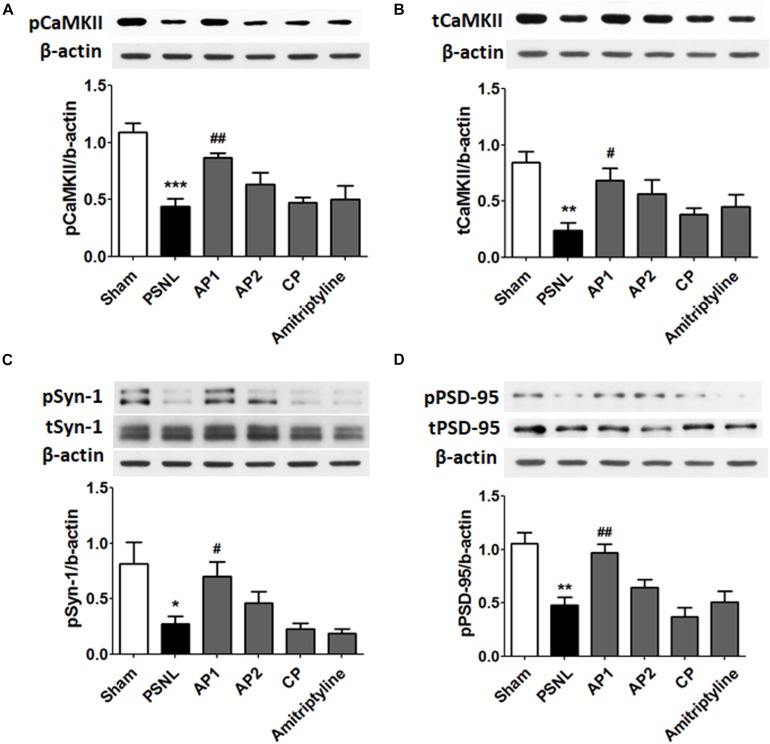
Effects of acupuncture on the expression levels of CaMKII and synaptic proteins. These results show the changes in protein levels of hippocampal CaMKII (**A,B**; *n* = 5/group) and synaptic proteins (**C,D**; *n* = 4/group) after administration of acupuncture (AP1, AP2, or CP) or amitriptyline (10 mg/kg, i.p.) for 28 consecutive days. ^∗∗^*p* < 0.01, ^∗∗∗^*p* < 0.001 compared to the Sham group. ^#^*p* < 0.05, ^##^*p* < 0.01 compared to the PSNL group. Data are expressed as the mean ± SEM. The same β-actin value was used **(A,C**; **B,D)**. AP1, acupuncture 1 (GB30 and GB34); AP2, acupuncture 2 (HT7 and GV20); CP, control point.

CaMKII and PKC-γ have a critical role in learning and memory functions. Their functions are regulated through Ca^2+^ influx mediated by NR2B receptors in post synaptic membranes. The reduction of CaMKII expression levels have been found in the hippocampus of neuropathic pain models (Xu et al., 2012). Therefore, we examined the effects of acupuncture (AP1, AP2, or CP) or amitriptyline (10 mg/kg, i.p.) treatments on the CaMKII expression levels in the hippocampus of PSNL mice. A one-way ANOVA showed a significant difference in proteins expression of pCaMKII and tCaMKII between the groups (each *F*_5,24_ = 10.66; *p* < 0.0001; *F*_5,24_ = 5.156; *p* = 0.0024), and Newman–Keuls *post hoc* tests showed that pCaMKII and tCaMKII expression levels were decreased in the PSNL group compared to the sham group (each *p* < 0.001, *p* < 0.01; Figures 7A,B). Notably, AP1 treatment significantly restored pCaMKII and tCaMKII expression levels (each *p* < 0.01, *p* < 0.05 vs. PSNL; Figures 7A,B). However, pCaMKII and tCaMKII protein expression levels were not improved in the AP2, CP and amitriptyline groups (Figures 7A,B). PKC-γ expression levels were not changed in the hippocampus by acupuncture treatment (Supplementary Figure 2).

### Effects of Acupuncture on Expression Levels of Synaptic Proteins in the Hippocampus in PSNL Mice

Finally, we examined the effects of acupuncture administration on synaptic proteins, such as Syn-1 and PSD-95. A one-way ANOVA showed a significant difference in proteins expression of pSyn-1 (*F*_5,18_ = 5.600; *p* = 0.0028) and pPSD-95 (*F*_5,18_ = 10.11; *p* < 0.0001) between the groups, and Newman–Keuls *post hoc* tests showed that PSNL of the sciatic nerve induced a significant decrease in pSyn-1 (*p* < 0.05) and pPSD-95 (*p* < 0.01) compared with sham-operated mice. Acupuncture administration significantly improved pSyn-1 and pPSD-95 protein levels (each *p* < 0.05, *p* < 0.01 vs. PSNL). However, pSyn-1 and pPSD-95 protein levels were not significantly altered in the AP2 and CP groups (Figures 7C,D). Our data showed that the acupuncture treatment restored impaired synaptic proteins in the hippocampus following neuropathic pain.

## Discussion

Chronic pain is characterized as having a multidimensional aspect, involving nociception and affective or cognitive procession. Acupuncture is known to have an analgesic effect and improve cognitive impairment in chronic pain patients (Paramore, 1997; Couilliot et al., 2013). However, the detailed mechanisms associated with the co-curation effects of acupuncture on pain and cognitive impairment are yet to be revealed. In the present study, we found that the acupuncture treatment at acupoints GB30 and GB34 improved both nociceptive behavior and cognitive impairment associated with a PSNL-induced neuropathic pain model. The acupuncture group was classified as a different one from the PSNL group when analyzed using a decision tree algorithm of machine learning. We also found that acupuncture can enhance synaptic plasticity through increasing LTP as well as expression levels of NR2B and GluR1 in the hippocampus, which were impaired in the PSNL group. In addition, the expressions of CaMKII and synaptic proteins such as pPSD-95 and pSyn-1, indicators of synaptic plasticity, were enhanced by acupuncture treatment.

Central sensitization that develops following peripheral nerve injuries is thought to contribute to chronic pain and the deleterious effects on cognitive functions in preclinical models and in patients (Schnurr and MacDonald, 1995; Nicholson and Verma, 2004; Kodama et al., 2007, 2011). Here, we observed that PSNL mice with prolonged mechanical allodynia had impairments in the working and recognition memory when subjected to Y maze and NOR tests, consistent with recent studies (Kodama et al., 2011; Dimitrov et al., 2014; Liu et al., 2017). In contrast, acupuncture treatment (AP1) resulted in the improvements of memory-function as well as mechanical allodynia. Other acupoints or non-acupoints did not result in such improvement, which implies that acupoints GB30 and GB34 are specific for these therapeutic effects. Next, a random decision forest classifier in machine learning was used to determine whether the therapeutic effects of acupuncture can be predicted based on the pain and cognitive behaviors. Machine learning classifiers are specific applications of machine learning technology that use individual features to predict the pre-assigned class to which a given example belongs (Breiman, 2001; Geurts et al., 2006). The success of a given model can be assessed by examining how faithfully it predicts group membership, also allowing for the comparison of performance between feature sets (Pedregosa et al., 2011). We found that the mice treated with acupuncture were not only classified as a different group from the PSNL mice, but also shifted toward the sham mice. In contrast, mice treated with acupuncture at the control points clustered with the PSNL group.

The hippocampus plays a critical role in cognitive functions. Decreased hippocampal volume and alteration of synaptic plasticity have been found in Alzheimer’s disease rodent models with cognitive impairment (Tozzi et al., 2015; Jin et al., 2017). Such hippocampal changes were also observed in chronic pain, and the reduced synaptic plasticity might be associated with the cognitive impairment comorbid with pain (Valet et al., 2009; Zimmerman et al., 2009; Mutso et al., 2012). LTP is a cellular model of activity-dependent changes in synaptic strength, underlying information storage and memory creation (Kim et al., 2001). Recently, the decreased hippocampal LTP was reported in several pain models such as the peripheral nerve injury-induced cognition-impaired mice (Kodama et al., 2011; Mutso et al., 2012; Chen et al., 2017; Liu et al., 2017). Consistent with previous studies, we also found that LTP was reduced when measured in the hippocampal CA1 of PSNL mice with cognitive impairment. Moreover, we found that acupuncture enhanced LTP compared to the PSNL-induced neuropathic pain mice. Recent experiments in chronic pain reported that acupuncture may modulate synaptic function and regulate cognitive impairment, but no molecular mechanisms have been proposed (Xu et al., 2012; Chen et al., 2017). Therefore, we investigated the underlying mechanisms associated with the improvements in cognitive function and pain by acupuncture treatment by focusing on the LTP-related molecular mechanisms in the hippocampus.

Induction and maintenance of LTP at the Schaffer collateral-CA1 synapses is NMDA and AMPA receptors-dependent (Holmes and Grover, 2006; Fonseca, 2012; Luscher and Malenka, 2012; Bliss and Collingridge, 2013). NR2B increases CaMKII activity by increasing Ca^2^^+^ influx into the synapse, which then increases the expression of GluR1 in the synapse and thus mediates many important brain functions including cognition, learning and memory (Tovar and Westbrook, 1999; Lisman et al., 2002; Goebel et al., 2005; Wang H. et al., 2015; Shang et al., 2017). The down-regulation of NR2B, GluR1, CaMKII and synaptic proteins in the hippocampal neurons are associated with learning-memory deficits and cognitive dysfunctions in Alzheimer’s disease (AD) animal models (Liu et al., 2016; Mariani et al., 2017; Zhu et al., 2018). As shown in a recent AD study, the expression of NR2B in the hippocampus was shown to be reduced in a PSNL-induced neuropathic pain model (Wang X.Q. et al., 2015). Zhu et al. reported that the expression of GluR1 as well as the neurons co-expressing brain-derived neurotrophic factor and GluR1 were downregulated in the hippocampal CA3 region of neuropathic pain mice, which was also correlated with the pain-related comorbid conditions (Zhu et al., 2017). Consistent with these studies, our results revealed that hippocampal NR2B and GluR1 were significantly downregulated in the CA1 and CA3 of the PSNL neuropathic pain model, whereas acupuncture treatment rescued them. Interestingly, the correlation analyses revealed that the expression levels of NR2B and GluR1 are positively correlated with the cognitive functions, while negatively correlated with pain behaviors. This implies that the increase in NR2B and GluR1 in the AP1 group can play a pivotal role for the restoration of pain and comorbid cognitive dysfunction. In addition, we showed that acupuncture enhanced the expression levels of pCaMKII and tCaMKII in the hippocampus, while they were decreased in a neuropathic pain model. These results are consistent with a recent study showing that acupuncture further reduces CaMKII expression level in the hippocampal CA3 following a peripheral nerve injury (Xu et al., 2012). Activation of CaMKII is crucial for LTP induction in the hippocampus, and it has been implicated in activity-dependent synaptic strengthening (Lisman et al., 2002). PKC-γ is activated through increased intracellular Ca^2+^ influx, but this was not improved by acupuncture administration. Fernández de Sevilla and Buño et al. suggested that CaMKII has an important role in the initiation of the acetylcholine-mediated enhancement of NMDAR- and AMPAR-mediated transmission, but that PKC might be essential in the initiation of the acetylcholine-mediated augment of only NMDAR-mediated transmission (Fernandez de Sevilla and Buno, 2010). Therefore, we postulate that the difference between CaMKII and PKC expression patterns in the hippocampus might be involved in cholinergic mechanisms on the acupuncture treatment, and further studies are required.

Finally, we demonstrated the significant alteration of synaptic proteins such as Syn-1 and PSD-95 in the hippocampus of the PSNL model mice. Syn-1 is a presynaptic terminal specific marker involved in transport, emissions, and recycling of the vesicles, and believed to play an important role in the process of the calcium-dependent neurotransmitter release, such as glutamate (Valtorta et al., 2004). PSD-95 is a structural protein of post-synapse and is increased by the activation of CaMKII, a protein that plays a crucial role in synaptic plasticity (Cheng et al., 2006). In this study, we found that acupuncture treatment recovered the decreased levels of phosphorylated Syn-1 and PSD-95 in the hippocampus of PSNL-induced neuropathic pain. Therefore, our results suggest that acupuncture can improve both nociception and cognitive impairment by regulating glutamate transmission through increasing the expressions of glutamate receptors and synaptic proteins.

It is well known that synapses between neurons are the structural basis of neural connection and neural plasticity. In the recent study, the total dendrite length, the number of dendrite branches, and spine densities in the basal and apical dendrites of CA1 pyramidal neurons were reduced significantly in the neuropathic pain mice (Liu et al., 2017). Therefore, further studies are needed to explore whether acupuncture can change the morphology of CA1 pyramidal neurons related to the synaptic plasticity.

The limitation of this study is that it is not known how changes in the peripheral nervous system affect the brain. Acupuncture showed analgesic effect through increases in adenosine and adenosine monophosphate release in the muscle, and this effect disappeared in the adenosine A1 receptor knockout mice (Goldman et al., 2010). The locally activated ERK signaling pathway was also mediated in the acupuncture analgesia (Park et al., 2014). However, since this study was focused on the CNS, we did not closely observe changes in adenosine, ATP or ERK pathway in the peripheral nervous system following acupuncture treatment. In addition, the stimulation of the metabotropic A1 receptors are known to be involved in modulation of chronic pain at the spinal and supra spinal level, in both neuronal and, at least partly, glial or microglial cells (Luongo et al., 2012; Luongo et al., 2014). Moreover, LTP in hippocampal CA1 area was inhibited in the adenosine A1 receptor knockout mice (Zhou et al., 2018). Further studies are needed to define the relationship between adenosine A1 receptors in the local tissue and glutamate receptors in hippocampus.

In conclusion, we demonstrated that acupuncture treatment can improve both allodynia and comorbid cognitive impairments simultaneously, and that the therapeutic effects of acupuncture were correlated with the increased expressions of NR2B and GluR1 in the hippocampus. Acupuncture also enhanced pCaMKII and tCaMKII as well as the pre- and post-synaptic proteins in this area. Finally, these molecular changes contribute to the increase of LTP in the CA1 of the hippocampus. These results might imply that acupuncture can be one of the potential options for controlling both pain and comorbid cognitive impairments, while further clinical trials are needed to prove it.

## Data Availability

All datasets generated for this study are included in the manuscript and/or the Supplementary Files.

## Ethics Statement

The animal study was reviewed and approved by Dongguk University Animal Care Committee for Animal Welfare (IACUC-2017-022-1). Written informed consent was obtained from the owners for the participation of their animals in this study.

## Author Contributions

J-HJ, E-MS, YR, M-YS, and H-JP conceived the experiments. J-HJ, J-YP, and H-JP contributed to the materials and tools. J-HJ, Y-KK, J-YO, W-MJ, H-KK, and H-YK performed the experiments. J-HJ analyzed and interpreted the data and wrote the main manuscript draft. J-HJ and H-JP designed the experiments and wrote, edited, and revised the manuscript. All authors had input into the manuscript and have approved the manuscript for publication.

## Conflict of Interest Statement

W-MJ was employed by company VUNO Inc. The remaining authors declare that the research was conducted in the absence of any commercial or financial relationships that could be construed as a potential conflict of interest.

## Footnotes

1 http://scikit-learn.org/stable/
