# Supplementary material for: Acupuncture Improves Comorbid Cognitive Impairments Induced by Neuropathic Pain in Mice
Source: Front Neurosci. 2019 Sep 20;13:995. doi: 10.3389/fnins.2019.00995 (PMC6763606; doi:10.3389/fnins.2019.00995)
Supplement: Supplementary file 1 [file Table_1.DOCX]

**Acupuncture Improves Comorbid Cognitive Impairments Induced by Neuropathic Pain in Mice**

Jae-Hwan Jang^1,2,3^, Yu-Kang Kim^1,2^, Won-Mo Jung^4^, Eun-Mo Song^5^, Hee-Young Kim^6^, Ju-Young Oh^1,2,3^, Ji-Yeun Park^7^, Mi-Yeun Song^5^, Hi-Joon Park^1,2,3*^

**Supplementary Material**


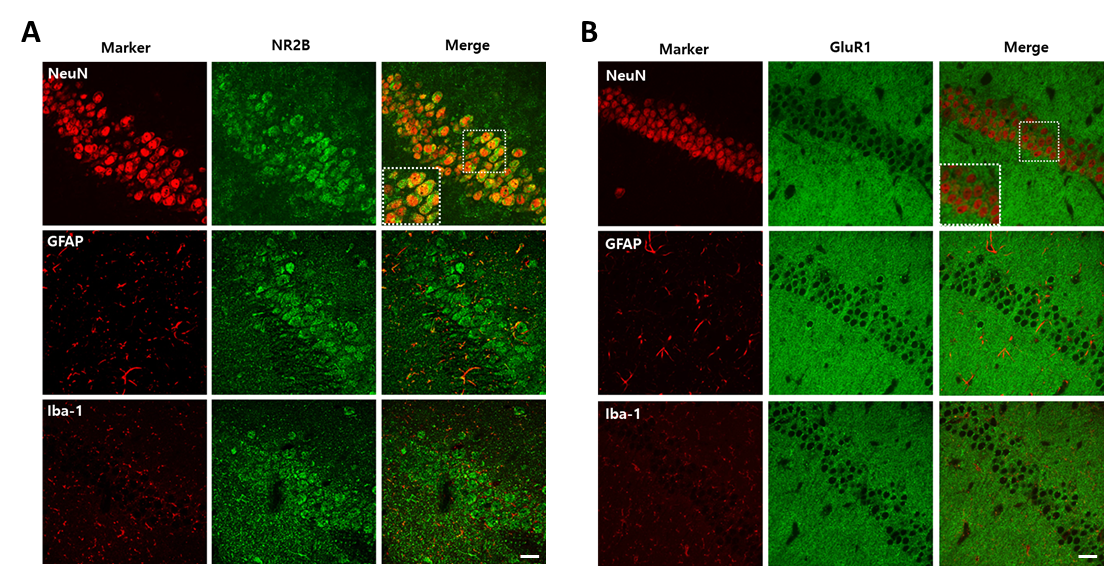


Supplementary Figure 1. The expression of hippocampal NB2B and GluR1 in neurons. Histological examinations of tissue sections from the hippocampus using immunofluorescence show the expression of NR2B (green; A), GluR1 (green; B) and NeuN (red; A and B). The insets (dotted square boxes) show NR2B-positive NeuN and GluR1-positive NeuN, respectively. Scale bar: 30 μm.


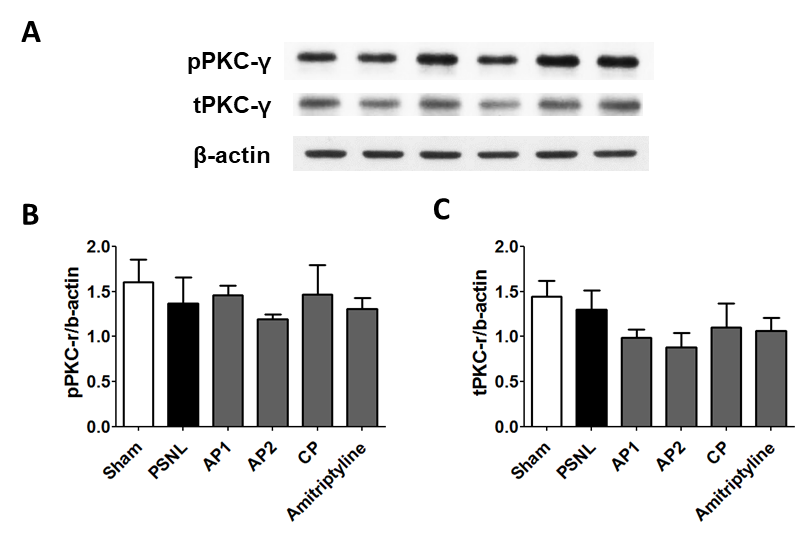


Supplementary Figure 2. The expression levels of PKC-γ protein in the hippocampus. These results show the changes in hippocampal PKC-γ protein levels after administration of acupuncture (AP1, AP2 or CP) or amitriptyline (10 mg/kg, i.p.) for 28 consecutive days (A-C). *n* = 4 in all groups.


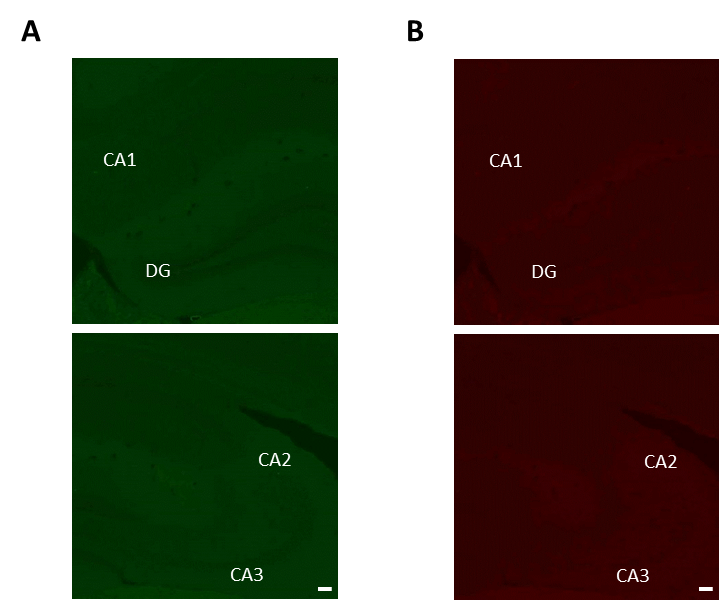


Supplementary Figure 3. Control images to show the specificity of the NR2B and GluR1 immunofluorescence in the hippocampus. Representative images show that the immunoreactivity of NR2B, GluR1 (green; A) and NeuN (red; B) were not found when the tissues were incubated with the antibody diluent alone without primary NR2B, GluR1 and NeuN antibodies. Scale bar: 100 μm.


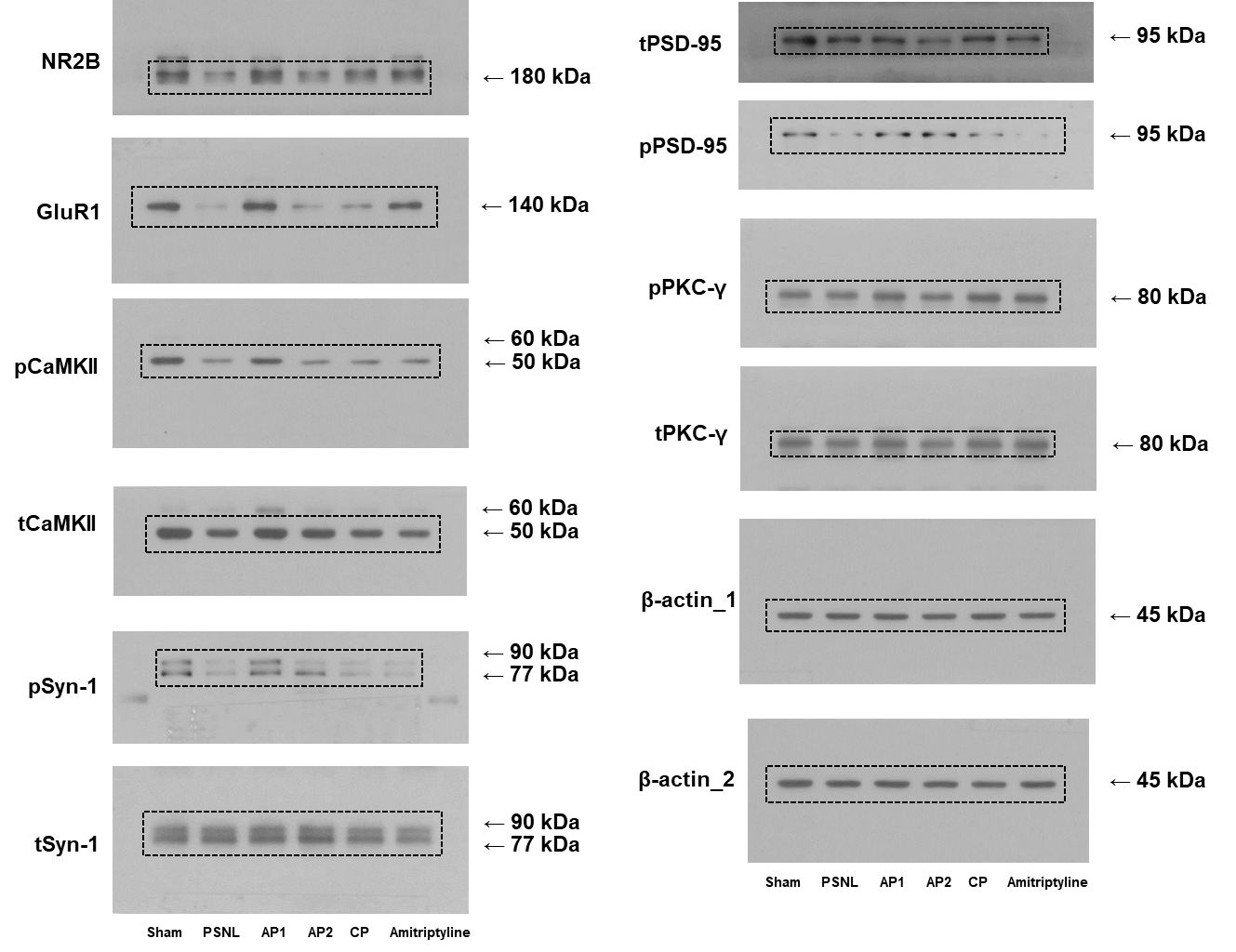


Supplementary Figure 4. Original scan of representative western blot analysis images in Fig. 5, 6, 7 and supplementary Fig. 2
